# Safety of medication use during pregnancy in mainland China: based on a national health insurance database in 2015

**DOI:** 10.1186/s12884-019-2622-y

**Published:** 2019-12-03

**Authors:** Jingyuan Zhang, Carolina Oi Lam Ung, Xiaodong Guan, Luwen Shi

**Affiliations:** 1 Department of Pharmacy Administration and Clinical Pharmacy, School of Pharmaceutical Sciences, Peking University, Beijing, China; 2 State Key Laboratory of Quality Research in Chinese Medicine, Institute of Chinese Medical Sciences, University of Macau, Macao, China; 3 Department of Population Medicine, Harvard Medical School and Harvard Pilgrim Health Care Institute, Boston, MA USA; 4 International Research Center for Medicinal Administration, Peking University, Beijing, China

**Keywords:** Medication use, Safety, Pregnancy, China, FDA pregnancy risk category

## Abstract

**Background:**

Medication safety during pregnancy has drawn global attention, little of which has been reported about the Chinese population. This study aims to describe patterns and risks of medication use among pregnant women in mainland China with reference to the U.S. Food and Drug Administration (FDA) pregnancy risk category.

**Methods:**

Hospital diagnostic and drug dispensing information of a national representative sample of basic medical insurance (BMI) beneficiaries was obtained from the China Health Insurance Association (CHIRA) database in 2015. Prevalence of use and number of medicines involved in each risk category were calculated. Most commonly used medicines from each risk category were illustrated. Factors associated with the use of category D/X medicines were evaluated through multiple logistic regression.

**Results:**

Out of 11,373 women who had singleton deliveries in 2015, there were 2896 women with records covering their entire pregnancies, 5377, and 7946 women with records through the 2nd, and the 3rd trimester, respectively. It was found that 11.1% pregnant women used at least one medication and a total of 321 medications had been used during pregnancy. Most pregnant women used medicines which were classified FDA category C (66.2%), followed by category B (57.8%), category A (16.8%), category X (7.5%) and category D (5.0%). The most commonly used medicines from category D and X were anxiolytics and hormonal preparations respectively. Women who were from mid-western area (*p* = 0.045) or used four or more medications (*p* < 0.001) were more likely to use category D/X medicines.

**Conclusions:**

This study revealed that about one in ten pregnant women used at least one medication during pregnancy in China and a significant number of them used FDA Category D or X medicines. The usage patterns identified in the present study indicate that sub-optimal medicine use might exist warranting further evaluation and intervention in future studies. More efforts are needed to uncover the safety concerns about medication use during pregnancy and improve current information system for clinical practice.

## Background

Medication safety during pregnancy has drawn constant attention globally since the thalidomide disaster in the 1960s [1]. Information on prenatal medication safety is seriously lacking, since pregnant women are usually excluded from pre-marketing clinical trials out of ethical concerns. Animal studies as a source of reference also have significant limitations and should be interpreted with cautions [2, 3]. To facilitate clinical decision-making, many countries have developed a classification system based on existing clinical and animal evidence to describe the risks of medication use during pregnancy, such as the Swedish system (Farmaceutiska Spesialiteter i Sverige, FASS), the U.S. Food and Drug Administration (FDA) pregnancy risk category, and the Australian Drug Evaluation Committee (ADEC) classification [4–6].

The FDA pregnancy risk category was developed in 1979, consisting of five categories (A, B, C, D, and X) based on published evidence of the risks and benefits of medication use during pregnancy [7]. According to the Requirements on Content and Format of Labeling for Human Prescription Drug and Biological Products (21 CFR Part 201) issued in 1979, there were two subsections under the “Pregnancy” part: (i) Teratogenic effects which refer mainly to the risk of fetal abnormalities related to medication use; (ii) Nonteratogenic effects which refer mainly to the drug’s effects on reproduction or nonteratogenic effects in the fetus or newborn infant such as withdrawal symptoms or hypoglycemia [8]. While highlighting the toxicological consideration of antenatal medication use, this system was criticized for its oversimplification and incompetence to guide clinical practice and was later replaced by a narrative labelling system known as “Pregnancy and Lactation Labeling Rule (PLLR)” in 2015 [4, 6, 9]. However, PLLR still does not provide a definitive “yes” or “no” answer on medication safety and cannot be used for retrospective chart review [10]. Despite certain limitations, the old FDA risk category can provide a rough description of medication use risks during pregnancy on a population basis [6, 9] and has been widely adopted in drug utilization studies both in developed and developing countries [10–15]. For the purpose of this study, the term “risks of medication use” will be used to cover both teratogenic and nonteratogenic effects which could be evaluated in the FDA pregnancy risk category [12, 16, 17].

Previous studies reported a prominent use of medicines in category A, B, and C, and a considerable prevalence of higher-risk medication use (category D/X) during pregnancy ranging from 1.6 to 8.2%, which again raised concerns about maternal medication use [12, 13, 17–21]. Age, insurance type, region, health status, physician specialty, and number of medications were found significantly associated with category D/X medicine use [17, 20]. However, too little is currently known about medication safety during pregnancy in mainland China.

As the number of live births reached 17.6 million in 2017 nationally [22], pregnant women in China comprise an increasingly larger population. Moreover, family-planning policy in China transitioned from one-child birth control to a universal two-child policy since 2016 [23], which has been reported to be associated with some changes of birth characteristics in China: more women being multiparous, and aged 35 or over at the time of delivery [24]. This presents greater challenge to clinical practice to the appropriateness and safety of medication treatment during pregnancy especially for pregnant women aged over 35 years or above [25]. In order to optimize maternal and infant health, research on maternal medication safety and its determinants is urgently needed to inform policy makers and health providers. Given there is no authoritative guidance on medication use during pregnancy officially issued in China, in this study, we aimed to (i) describe the patterns and risks of medication use with reference to FDA pregnancy risk category among pregnant women derived from a national health insurance database in 2015 in China and (ii) evaluate the factors associated with the use of medicines with potential harms during pregnancy (FDA category D/X).

## Methods

### Data source

By the end of 2015, over 1.33 billion (97%) of Chinese population was covered by three public insurance schemes, Urban Employee Basic Medical Insurance (UEBMI) mandatory for urban employees, Urban Resident Basic Medical Insurance (URBMI) targeting the unemployed, children, students, and the disabled in urban areas, and New Rural Cooperative Medical Scheme (NRCMS) for rural residents [26]. Medical service utilization information of every UEBMI and URBMI (hereafter jointly referred to as BMI) beneficiary in hospitals and BMI designated drugstores is routinely collected by BMI databases at city level, which includes: (1) BMI beneficiaries’ demographic information, e.g., unique personal identifiers, birth date, and gender; (2) inpatient and outpatient medical encounters information, e.g., principal diagnosis for each encounter coded with International Classification of Diseases 10th Revision (ICD-10), admission and discharge dates, and facilities’ information; (3) all medical service utilization data for each encounter, e.g. the name, date, price, and quantity of medications dispensed and procedures performed, regardless of whether or not these items are covered by current BMI schemes.

The China Health Insurance Association (CHIRA) employed a two-stage systematic sampling design to obtain a national representative sample of BMI beneficiaries and extracted cross-sectional medical service utilization data annually from these city-level BMI databases, which forms the unique national health insurance database known as the CHIRA database [27]. Medication utilization data of pregnant women in this study was obtained from the CHIRA database in 2015, which overall contained information on 37.3 million BMI beneficiaries sampled from 82 cities nationwide, representing about 2% of the total population in Mainland China. Since the CHIRA data was de-identified, this study was exempt from Institutional Review Board review by the Ethics Committee of Peking University Health Science Center, Beijing, China (No. IRB00001052–17022).

### Study population

Women aged 12–54 with a singleton delivery between January 1st 2015 and December 31th 2015 in the national health insurance database were identified by ICD-10. As direct measures of gestational age such as the last menstrual period (LMP) were lacking in the database, in order to pinpoint each period of medication use, we employed a delivery-date algorithm validated by Li Q. et al. (2013) assuming a gestational duration of 270 days (245 days for preterm delivery), with three 90-day trimesters during pregnancy (65 days of the third trimester for preterm delivery) based on admission date for delivery [28]. Only women who had a singleton delivery in 2015, were between 12 and 54 years old at delivery, and whose records in the CHIRA database covered at least one trimester were included in this study.

### Medication information

According to the approval number issued by National Medicinal Product Administration (NMPA), medications were divided into modern Western medicine and Traditional Chinese Medicine (TCM). As FDA pregnancy risk category was only applicable to Western medicine, TCM were excluded from analysis in this study. Records with missing information were also excluded from the analysis.

### FDA pregnancy risk category information

Information on FDA pregnancy risk category (A, B, C, D and X) was identified from the MicroMedex database and the Monthly Index of Medical Specialties (MIMS) database. If a medication was identified as compound formula, the category of the ingredient with the highest risk was taken into analysis. Medications without available FDA pregnancy category information were classified as unknown. With available evidence indicating potential fetal abnormalities, medications in category D (positive evidence of human fetal risk and should only be used in a life-threatening situation or lack of alternatives) or category X (contraindicated in pregnant women as the risk clearly outweighs any possible benefit) [29] were usually regarded as medications with potentially harmful for pregnant women in present studies [17, 21], and in this study as well.

### Outcome measures and statistical analysis

To summarize medication use patterns during pregnancy, for each trimester and through the entire pregnancy, prevalence of overall medication use was defined as the proportion of women with any medication dispensed. Prevalence for specific medication use was calculated as the percentage of women with a specific medication dispensed to all women with at least one medication dispensed in each trimester and during the entire pregnancy. Prevalence in each risk category was displayed as the proportion of women exposed to medications under a specific risk category among all the women with at least one medication dispensed in the time frame of interest. Among women with at least one medication dispensed in each trimester and during the entire pregnancy, we calculated total number of different medications dispensed under each FDA risk category by generic names.

Descriptive analyses was performed on sample characteristics, medication use prevalence, and overall distribution of medication use risks. Numerical variables were summarized with median while categorical variables with proportions. We used Chi-square test to examine prevalence difference among three trimesters. Risk factors associated with high-risk medication use (FDA D/X category) were explored using multiple logistic regression with odds ratio (OR) and 95% confidence intervals (95% CI).

## Results

### Sample characteristics

**Fig. 1:**
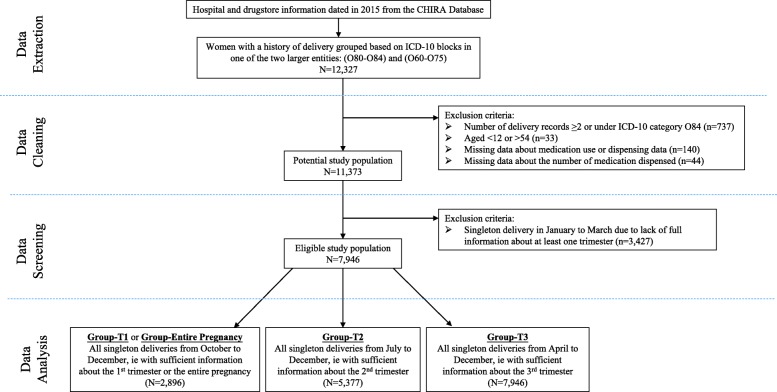
Flow diagram of sample selection process. CHIRA: China Health Insurance Association; ICD-10: the International Classification of Diseases 10th Revision

In total, 11,373 women aged from 12 to 54 with a singleton delivery in 2015 were identified from the CHIRA database. According to our estimates of each gestational period, 7946 women had records that covered at least one trimester. Among them, there were 2896, 5377, and 7946 women with records covering the 1st, 2nd, and 3rd trimester, respectively (Fig. 1, Additional file 1). Most women aged between 25 and 29 years old. The majority lived in eastern area, participated in URBMI, and had a natural delivery (Table 1).

**Table 1:** Sample characteristics in each period

	Gestational stages		
	T1/ Entire pregnancy	T2	T3
Total	2896	5377	7946
Age, *n* (%)			
< 25	646 (22.3%)	1128 (21.0%)	1607 (20.2%)
25–29	1282 (44.3%)	2393 (44.5%)	3601 (45.3%)
30–34	688 (23.8%)	1316 (24.5%)	1935 (24.4%)
≥ 35	280 (9.7%)	540 (10.0%)	803 (10.1%)
Area, *n* (%)			
Eastern	1621 (56.0%)	3235 (60.2%)	4833 (60.8%)
Mid-western	1275 (44.0%)	2142 (39.8%)	3113 (39.2%)
Insurance type, *n* (%)			
URBMI	2170 (74.9%)	3947 (73.4%)	5833 (73.4%)
UEBMI	726 (25.1%)	1430 (26.6%)	2113 (26.6%)
Deliver type, *n* (%)			
Natural delivery	1795 (62.0%)	3362 (62.5%)	4981 (62.7%)
Other	1101 (38.0%)	2015 (37.5%)	2965 (37.2%)

*URBMI* Urban Resident Basic Medical Insurance

*UEBMI* Urban Employee Basic Medical Insurance

### Overall medication use in each risk category during pregnancy

Overall, 11.1% of sampled women had used at least one medication during pregnancy and 321 different western medicines used by these women were identified. The most commonly used medicines were in FDA category C (66.2%) and B (57.8%), followed by category A (16.8%). Only less than one tenth of the women with at least one medication dispensed used medicines with higher risks (category X: 7.5%, D: 5.0%). The variety of medicines used in each trimester increased from the first trimester (*n* = 171) to the third trimester (*n* = 318). Most medicines were assigned in FDA category C (38.3%) and B (20.9%), only a few fell in categories with higher risks (category D: 5.9%, X: 3.7%), and category A held the least number of medications. Besides the above, there were still a substantial proportion of pregnancies exposed to medicines without an assigned FDA risk category (41.9–49.4%), accounting for nearly 30% of all medications identified in this study (Table 2).

**Table 2:** prevalence and number of medications used by FDA risk category during pregnancy and each trimester

	Gestational stages			*p* value^*a*^	Entire pregnancy
	T1	T2	T3		
Overall prevalence, %	5.4	4.5	6.0	0.001 **	11.1
Prevalence of each category among women with medication use, % ^*b*^					
A	17.4	15.6	10.9	0.057	16.8
B	61.9	37.7	34.2	< 0.001***	57.8
C	38.7	68.9	72.8	< 0.001***	66.2
D	3.2	2.9	3.1	0.973	5.0
X	5.8	2.1	6.3	0.042 *	7.5
Unknown	41.9	43.0	46.3	0.531	49.4
Total number of different medications, *n*	171	227	318		321
Number of different medications in each category, *n* (%) ^*c*^					
A	6 (3.5)	7 (3.1)	8 (2.5)	0.814	8 (2.5)
B	40 (23.4)	54 (23.8)	71 (22.3)	0.916	67 (20.9)
C	68 (39.8)	87 (38.3)	132 (41.5)	0.753	123 (38.3)
D	6 (3.5)	8 (3.5)	12 (3.8)	0.984	19 (5.9)
X	8 (4.7)	3 (1.3)	8 (2.5)	0.117	12 (3.7)
Unknown	43 (25.2)	68 (30.0)	87 (27.4)	0.562	92 (28.7)

^*a*^The P value of comparison tests among three trimesters. * *p* < 0.05; ** *p* < 0.01; *** *p* < 0.001

^*b*^Each pregnant woman could be exposed to more than one FDA category

^*c*^Columns might not add to 100% because of rounding error

### Most commonly used medicines by FDA risk category

Of all the medicines used during pregnancy and each trimester, Additional file 2 summarized the top 20 most common ones in FDA category A, B, and C. Irrigating solutions for injection or infusion, progesterone, vitamins, systematic hormonal preparations, and antibiotics were used frequently during pregnancy and each trimester.

As shown in Table 3, there were still some women exposed to medicines which had relatively higher risks (D/X category) during pregnancy, more often in the 1st trimester and the 3rd trimester. Within category D, medicines for nervous system such as benzodiazepines (BZDs) like diazepam (0.93%), alprazolam (0.62%), and anti-epileptics like phenobarbital (0.31%), were the most common ones. Medicines classified as category X mainly included hormonal preparations such as oxytocin (3.11%) and misoprostol (0.93%). The use of antiviral drug ribavirin (1.55%), a category X medicine, was also prominent. In addition, early use of high-risk dermatological agents such as tretinoin (category D) and isotretinoin (category X) were also observed in this study.

**Table 3:** The most common FDA Category D/X medicines used by women who used at least one medication during pregnancy (%)

Generic name	Gestational stages			Entire pregnancy
	T1	T2	T3	
FDA category D				
Diazepam	/	0.41	0.84	0.93
Irbesartan	0.65	0.82	0.42	0.62
Alprazolam	/	/	0.42	0.62
Phenobarbital	0.65	/	0.21	0.31
Methimazole	0.65	/	/	0.31
Midazolam	0.65	/	/	0.31
Tamoxifen	0.65	/	0.21	0.31
Tretinoin	0.65	/	/	0.31
Amikacin	/	0.41	0.42	0.31
Mycophenolic acid	/	0.41	0.21	0.31
Valsartan	/	0.41	0.21	0.31
Gentamycin	/	/	0.42	0.31
Doxycycline	/	0.41	/	0.31
Lorazepam	/	/	0.21	0.31
Lithium	/	/	0.21	0.31
FDA category X				
Oxytocin	1.29	0.41	4.19	3.11
Ribavirin	/	1.23	1.05	1.55
Misoprostol	0.65	/	0.84	0.93
Chorionic gonadotrophin	1.29	/	/	0.62
Mifepristone	0.65	/	0.21	0.62
Goserelin	0.65	/	/	0.31
Clomifene	0.65	/	/	0.31
Medroxyprogesterone	0.65	/	/	0.31
Isotretinoin	0.65	/	/	0.31
Estradiol Valerate	/	0.41	0.42	0.31
Valproic acid	/	/	0.21	0.31

*FDA* The U.S. Food and Drug Administration

### Factors associated with FDA category D/X medication use among women with at least one medication use during pregnancy

The results of multiple Logistic regression exploring factors related to the use of category D/X medicines among women with at least one medication use during the entire pregnancy were shown in Table 4. Women who were from mid-western area (*p* = 0.045), used four or more medications during pregnancy (*p* < 0.001), or those who regularly went to tertiary hospitals (*p* = 0.076) were more likely to use category D/X medicines compared with their counterparts.

**Table 4:** Factors associated with FDA category D/X medication use

Variables	Odds Ratio	95% CI	*p* value
Age	1.04	0.98–1.11	0.174
Area			
*Eastern*	*referent*	*referent*	*referent*
Mid-western	2.54	0.99–6.21	0.045*
Insurance type			
*URBMI*	*referent*	*referent*	*referent*
UEBMI	0.70	0.29–1.68	0.425
Deliver type			
*Natural delivery*	*referent*	*referent*	*referent*
Others	1.00	0.44–2.14	0.998
Level of mostly visited hospital			
*Secondary hospital or below*	*referent*	*referent*	*referent*
Tertiary hospital	2.10	0.94–4.91	0.076
Number of different medications used			
*< 4*	*referent*	*referent*	*referent*
≥4	4.17	2.01–9.11	< 0.001 ***

*FDA* The U.S. Food and Drug Administration

*URBMI* Urban Resident Basic Medical Insurance

*UEBMI* Urban Employee Basic Medical Insurance

*p < 0.05, ***p < 0.001

The p value of mid-western area (0.045) and used four or more medications ( < 0.001) were deemed significant and thus set in boldface

## Discussion

In this study, we found that about one in ten pregnant women reported use of at least one medication during pregnancy in China and categories C, B, and A medicine were most commonly used. While the overall prevalence of medication use is comparatively lower than what has been previously reported in other countries including Europe, North and South America and Australia (ranged from 50.0% to 81.2%) [30, 31], the use of higher-risk medications (category D 5.0%, category X 7.5%) were comparable with previous studies from the United States (category D 4.8–6.4%, category X 2.9–4.6%) [17, 32], and Canada (category D 5.2–7.3%, category X 2.5–8.2%) [21, 33, 34], and even more serious than those reported in Ireland (category D 2.5%, category X 3.2%) [18], the United Kingdom (category D 1.6%, category X 1.0%) [16], India (category D 1.55%, category X 0.17%) [12], and North Ethiopia (category D 0.5%, category X 0%) [13]. Great disparities in the study design and context, such as data sources, time period, inclusion criteria, variety of medicines on the market and its availability to the target population make it hard for direct comparisons internationally. Nevertheless, the results of the present study still revealed that a significant number of women were exposed to potentially harmful medicines (category D/X) warranting closer attention.

As for medicines with higher risks during pregnancy, we found that the most commonly used medicines in category D were mainly anxiolytics and anti-epileptics. Category X medicines including hormonal preparations such as contraceptives and fertility drugs were also prevalent. Similar results were reported in some previous studies with a nuance of the selection of specific medicines [11, 17, 34]. Such pattern was most likely due to the routine medicine use in unidentified early pregnancy. Nevertheless, it is important to note that this may not be interpreted as actual risks, since contraceptives and fertility drugs were classified as category X only because they were not needed during pregnancy rather than their teratogenic risks [6]. Meanwhile, an uneven distribution of medication use prevalence among three trimesters was also observed, as use of high-risk medicines mostly occurred in the first and third trimester. Given the fetus is most susceptible to damage from teratogens during the first trimester [2], the use of D/X medicines in the first trimester may result from unidentified or unplanned pregnancy of the early stage. These findings above were in accordance with many previous studies [18, 21, 32, 34, 35], indicating a similar pattern of higher-risk medication use during pregnancy across countries.

As widely acknowledged, medicines should be avoided during pregnancy as much as possible. However, use of tretinoin and isotretinoin in early stage during pregnancy had been identified in this study. These agents were mainly used for acne treatment and should have been avoided by pregnant women [36, 37]. Such usage indicates a potential gap between the clinical principle and practice. On the other hand, when withholding necessary treatments may place the pregnant women and their fetus in greater danger, it is important to balance the benefits with risks based on the patient’s concrete condition especially in the cases whereby safer alternatives are available [38, 39]. It was also observed that ribavirin, a medication contraindicated in women who are or may become pregnant due to its embryocidal and teratogenic effects in animals [40, 41], was one of the most commonly used category X medicines in this study. The use of ribavirin could and should be substituted by other options with lower risks during pregnancy (e.g. sofosbuvir in category B or oseltamavir in category C) [40, 42]. Apart from the choice of treatment, it is also crucial to take the dosage and the timing of medication use into consideration when evaluating the risks. As the most commonly used category D medicines, the prevalence of BZDs use for anxiety disorders was more prominent in the third trimester in this study. According to American College of Obstetricians and Gynecologists (ACOG) Guidelines, BZDs may lead to floppy syndrome and withdrawal syndromes in infants when maternal use occurred shortly before delivery [43]. These identified usage patterns in the present study indicates that sub-optimal medication use might exist which warrants further evaluation and intervention in future studies.

Results of Logistic regression showed that pregnant women who lived in mid-western area, used four or more medications, or regularly visited tertiary hospitals were more likely to be exposed to medications with higher risks (category D/X), which was similar to the findings from previous studies [18, 20, 44–46]. This was possibly because women in mid-western area of China tended to have a lower income level and poorer access to medical services, which makes them use medicines only when really necessary or under more serious circumstances, thus more likely to be exposed to high-risk medications. Also, women paying frequent visit to higher level hospitals or used more medications during pregnancy may be those with severe conditions, thus were more likely to be exposed under high-risk medications.

To the best of our knowledge, this is the first study that investigates the prevalence and patterns of medication use and risks during pregnancy according to FDA pregnancy risk category in mainland China. We used CHIRA database with electronic records of sampled population, which can be considered an “extended health insurance data” that contains information on all medicines dispensed to BMI beneficiaries, regardless of whether or not these medications are covered by current BMI schemes or whether they are prescription medications or over-the-counter products (OTC). The employment of the CHIRA database enabled a more accurate and reliable assessment of medication use, which may become an important source to advance Chinese population-based research and facilitate the integration of real-world evidence in clinical practice and local policy making.

However, there are several limitations to this study. First, our findings may not be representative of population in rural area, since CHIRA database mainly covers urban BMI beneficiaries. Second, our analysis did not include pregnancies ending up with miscarriages, terminations of pregnancy, or stillbirths, thus may carry the risk of underestimating the extent of maternal medication use, especially for potentially harmful medication use which may lead to negative pregnancy outcomes. Third, almost half of pregnant women with medication dispensed in this study used medications which were not assigned to any FDA category. Such unspecified risks may be mistaken as no risks and create a false impression of comfort. In addition, since the FDA category only covers Western medicine, the potential risks related to TCM use merits further investigation. Fourth, since only a limited number of variables were captured in the CHIRA database, we were unable to fully assess the association of other maternal characteristics with exposure to medications with higher risks, such as parity, complication of chronic disease and so on. In addition, since direct measures of gestational age were lacking in the database, our employment of the validated gestational age algorithm might still result in a small proportion of misclassification [47]. Finally, as mentioned above, this study only provides a rough sketch of the risks of medication use during pregnancy at a population level. Clinical consideration on the appropriateness and safety of antenatal medicine use requires further evaluation on a case-by-case basis.

## Conclusions

This study revealed that about one in ten pregnant women used at least one medication during pregnancy in China and a significant number of them used FDA Category D or X medicines. Anxiolytics and hormonal preparations were the most commonly used medicines classified as category D and X respectively. Pregnant women who were from mid-western area or used four or more medications were more likely to use medicines of higher risks. More efforts are needed to uncover medication safety during pregnancy and improve current information system for clinical practice.

## Supplementary information

**Additional file 1.** Detailed information of sample extraction process.

**Additional file 2.** Prevalence of most commonly used medications (top 20) in FDA category A, B, and C among women who used at least one medication during pregnancy (%).

## Data Availability

The data that support the findings of this study are available from China Health Insurance Association (CHIRA) but restrictions apply to the availability of these data, which were used under license for the current study, and so are not publicly available. Data are however available from the authors upon reasonable request and with permission of CHIRA.

## Abbreviations

BZDs: Benzodiazepines
CHIRA: China Health Insurance Association
FDA: U.S. Food and Drug Administration
ICD-10: International Classification of Diseases 10th Revision
NMPA: National Medicinal Product Administration
NRCMS: New Rural Cooperative Medical Scheme
TCM: Traditional Chinese Medicine
UEBMI: Urban Employee Basic Medical Insurance
URBMI: Urban Resident Basic Medical Insurance
