# Supplementary material for: Safety of medication use during pregnancy in mainland China: based on a national health insurance database in 2015
Source: BMC Pregnancy Childbirth. 2019 Dec 3;19:459. doi: 10.1186/s12884-019-2622-y (PMC6892234; doi:10.1186/s12884-019-2622-y)
Supplement: Supplementary file 1 — Additional file 1. Detailed information of sample extraction process. [file 12884_2019_2622_MOESM1_ESM.ppt]

## Slide 1
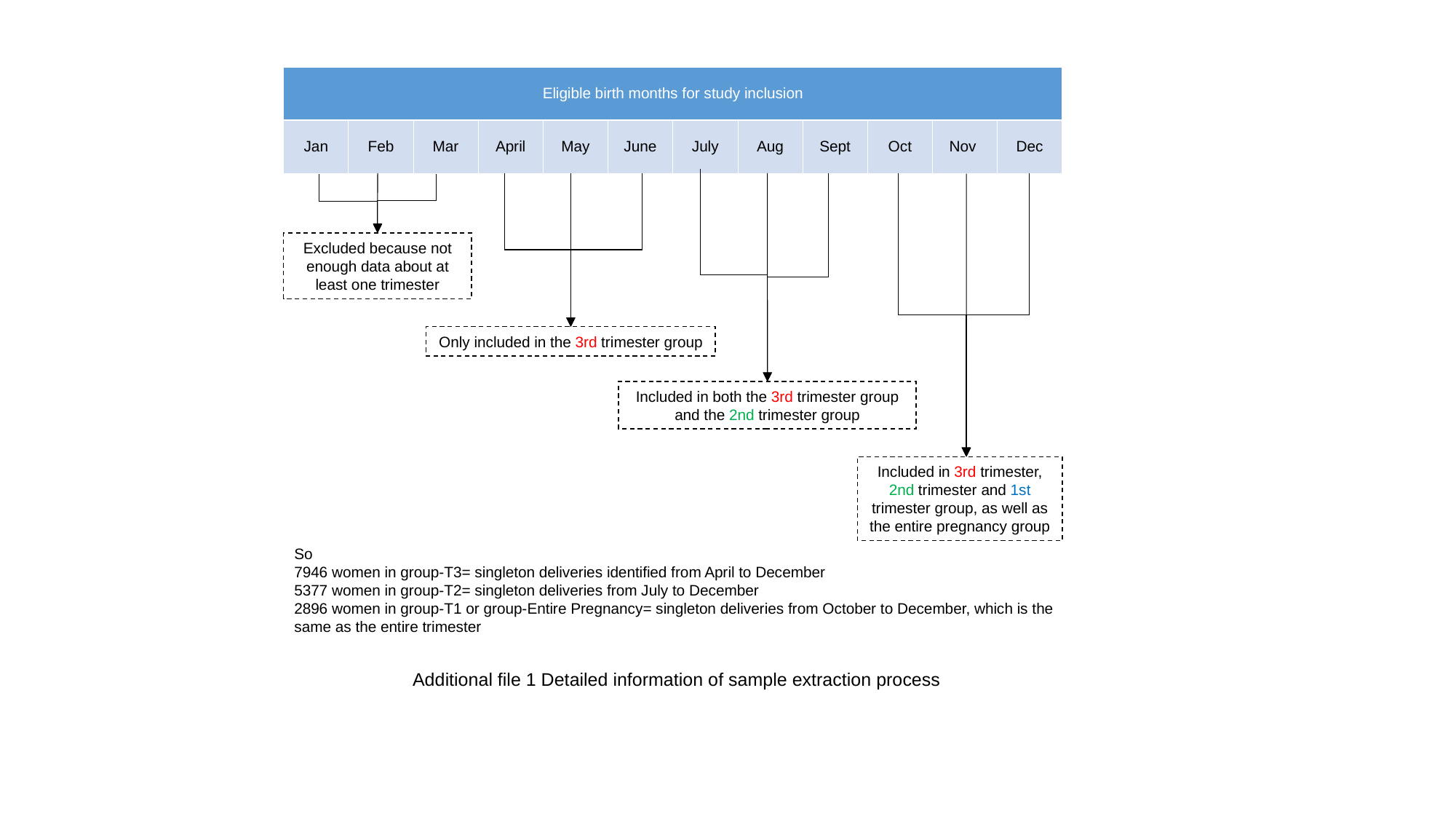

| Eligible birth months for study inclusion | | | | | | | | | | | |
| --- | --- | --- | --- | --- | --- | --- | --- | --- | --- | --- | --- |
| Jan | Feb | Mar | April | May | June | July | Aug | Sept | Oct | Nov | Dec |
Excluded because not enough data about at least one trimester
Only included in the 3rd trimester group
Included in both the 3rd trimester group and the 2nd trimester group
Included in 3rd trimester, 2nd trimester and 1st trimester group, as well as the entire pregnancy group
So
7946 women in group-T3= singleton deliveries identified from April to December
5377 women in group-T2= singleton deliveries from July to December
2896 women in group-T1 or group-Entire Pregnancy= singleton deliveries from October to December, which is the same as the entire trimester
Additional file 1 Detailed information of sample extraction process
