# Supplementary material for: Safety of medication use during pregnancy in mainland China: based on a national health insurance database in 2015
Source: BMC Pregnancy Childbirth. 2019 Dec 3;19:459. doi: 10.1186/s12884-019-2622-y (PMC6892234; doi:10.1186/s12884-019-2622-y)
Supplement: Supplementary file 2 — Additional file 2. Prevalence of most commonly used medications (top 20) in FDA category A, B, and C among women who used at least one medication during pregnancy (%). [file 12884_2019_2622_MOESM2_ESM.docx]

Additional file 2 Prevalence of most commonly used medications (top 20) in FDA category A, B, and C among women who used at least one medication during pregnancy (%)

| Generic name | Gestational stages | | | Entire pregnancy | FDA risk category |
| --- | --- | --- | --- | --- | --- |
|  | T1 | T2 | T3 |  |  |
| Glucose | 10.3 | 28.3 | 28.7 | 30.4 | C |
| Sodium chloride | 13.6 | 17.6 | 21.4 | 25.2 | C |
| Progesterone | 43.9 | 5.7 | 2.1 | 24.8 | B |
| Ascorbic acid (Vit. C) | 7.7 | 13.1 | 16.6 | 18.0 | C |
|  |  |  |  |  |  |
| Calcium Carbonate + Cholecalciferol (Vit. D3) | 2.6 | 20.5 | 14.5 | 10.6 | C |
| Dexamethasone | 1.3 | 2.5 | 9.2 | 9.3 | C |
| Potassium chloride | 6.5 | 6.6 | 3.6 | 8.7 | C |
| Glucose + Sodium chloride | 3.9 | 4.1 | 3.8 | 8.1 | C |
| Sterile Water | 3.2 | 0.8 | 6.9 | 7.5 | C |
| Pyridoxine (Vit. B6) | 4.5 | 6.2 | 2.9 | 6.8 | A |
|  |  |  |  |  |  |
| Magnesium sulfate | / | 7.0 | 9.0 | 6.5 | C |
| Sodium chloride complex | 3.9 | 2.5 | 3.1 | 5.0 | C |
| (Sodium chloride + Potassium chloride + Calcium chloride) |  |  |  |  |  |
| Amoxicillin | 2.6 | 3.3 | 1.7 | 4.7 | B |
| Levothyroxine | 3.9 | 5.7 | 5.9 | 4.4 | A |
| Amino acids | 2.6 | 1.2 | 2.7 | 4.0 | C |
| Lactated Ringers solution | 1.3 | 1.6 | 3.6 | 4.0 | C |
| (Sodium Lactate, Sodium chloride + Potassium chloride + Calcium chloride) |  |  |  |  |  |
| Azithromycin | 3.2 | 1.6 | 1.9 | 3.7 | B |
| Cefuroxime | 0.7 | 3.7 | 4.6 | 3.7 | B |
| Tocopherol (Vit. E) | 6.5 | 2.9 | 0.4 | 3.4 | A |
|  |  |  |  |  |  |
| Lidocaine | 1.3 | 2.5 | 2.9 | 3.4 | B |
| FDA: the U.S. Food and Drug Administration | | |  |  |  |
